# Music in the Treatment of Children and Youth with Prolonged Disorders of Consciousness

**DOI:** 10.3389/fpsyg.2016.00202

**Published:** 2016-02-17

**Authors:** Jonathan Pool, Wendy L. Magee

**Affiliations:** ^1^Harrison Research Centre, The Children's TrustTadworth, UK; ^2^Music Therapy Program, Boyer College of Music and Dance, Temple UniversityPhiladelphia, PA, USA

**Keywords:** music, treatment, children and youth, disorders of consciousness, review

## Introduction to the population

Children and youth with disorders of consciousness (DOC) are defined as those under 18 years of age who show wakefulness, but with absent or reduced awareness. This condition is considered to be prolonged when this state lasts for longer than four weeks. Hence, the term prolonged disorders of consciousness (PDOC) (Royal College of Physicians, 2013). Children and youth with DOC need care that can meet their highly complex needs. This care includes careful stimulation to elicit purposeful responses in assessment and evaluation, and managing an individual's environment optimally to meet their sensory needs. Accuracy in determining awareness is paramount due to several factors. First, ethical issues surround the provision of appropriate care (Ashwal, 2013) regarding the design and use of the type of sensory stimulation and the intensity of the intervention. Second, admission to rehabilitation programmes is affected by accurate diagnosis (Eilander et al., 2005), as this would ensure that those who could benefit are not excluded from admission to these programmes. Third, end-of-life decisions are critically dependent upon correct diagnosis (Ashwal and Cranford, 2002), when clinicians, families, and the legal system consider continuation or withdrawal of intervention in the light of the patients' pain and suffering and their prognosis for recovery.

Although guidance for working with adults with PDOC is available (Royal College of Physicians, 2013), there are no specific clinical guidelines for working with children and youth with PDOC. Recovery following brain injury in adults is better understood than in pediatric populations (Anderson and Yeates, 2010; Ponsford, 2013). This has resulted in theories about recovery of consciousness being based on adult brains, despite neurodevelopmental differences between child, and adult brains (Ashwal and Cranford, 2002; Perner and Dienes, 2003), particularly within the frontal lobes (Nicholas et al., 2014). This poses several problems for clinicians. Evaluation and treatment guidelines for rehabilitation with pediatric PDOC are adapted from those used with adults. However, guidance on adaptation is limited and dependent on clinicians' specialist knowledge, which is likely to be highly variable. The dearth of knowledge regarding neurological recovery from PDOC in childhood positions some theorists to argue that the immature brain is less susceptible to damage due to its plasticity, whereas others propose that the developing brain is more vulnerable to injury (Bower and Shoemark, 2012). Hence, more must be understood about recovery from brain injury in the pediatric population.

## Sensory stimulation with pediatric DOC

Sensory stimulation activates affected neural networks, maximizing the potential for neural reorganization through brain plasticity (Di and Schnakers, 2012; Pham et al., 2014). Sensory stimulation programs provide enriched environments that optimize stimulation to encourage experience-dependent changes to the brain at neuroanatomical and biochemical levels (Renner and Rosenzweig, 1987; Nithianantharajah and Hannan, 2006; Schreiber et al., 2014). When working with children and youth with PDOC, developmental factors must be considered. These include pre-morbid neurological development that influences the acquisition of fundamental skills in motor functioning and cognitive functioning, for example attention, memory, choice-making, and command-following capabilities. There are also significant differences in comprehension and expressive language development across ages, and these have important implications for the presentation of verbal and written commands as well as expectations of the patient's vocalizations. Educational level may also be important to consider when working with children and youth with PDOC, as this can also influence cognitive functioning and language development. Neurological changes related to emotional development and the importance of attachment at different stages must also be considered. The presence of parents can have a significant impact on children's anxiety and emotional states, and children with PDOC have been known to respond more effectively to parental voice above other voices (Machado et al., 2007). A final point to consider is that life/cultural experiences may have influenced the memories and templates that have been stored at earlier stages of development. So, specific life or cultural experiences could provide useful sources of stimuli for assessment and treatment of children and young people with PDOC. The developmental factors described above should inform clinical decisions about the stimuli used to elicit responsiveness and methods of presentation (Amari et al., 2012). A child's developing sensory processing system might demand the use of stimulation that raises arousal sufficiently but does not overwhelm the sensory or cognitive systems that process this information. A number of studies have discussed the use of music as a sensory stimulus with children with DOC. The suggested benefits include the provision of enriched environments through increasing arousal and maximizing the patients' potential to respond through supporting opportunities for fundamental interpersonal interaction (Bower et al., 2014). Researchers have postulated that the use of familiar songs in music therapy intervention compared with unfamiliar songs reduces agitation and cognitive load on the patients by increasing organization and orientation. This is supported by other research that suggests that familiar songs may reduce agitation and enhance orientation in adults suffering from post-traumatic amnesia (Baker, 2001). Presented live (rather than in recorded format), the use of familiar songs provides opportunities and invitations for interaction and development of social integration (Bower and Shoemark, 2012).

The loudness, number of instruments/voices, mood, tempo, and rhythmic complexity, and the meaning/familiarity of the music to the patient might all be key factors in providing optimal stimulation. Music, as a stimulus, can be manipulated using fine changes across wide ranges in intensity, complexity, and other musical parameters. As pre-linguistic children are biologically predisposed to attend to the musical elements of speech prosody, such as melodic contour and rhythmic patterning, these elements can be emphasized in musical interactions with the non-verbal typically developing or brain-injured child (Bower and Shoemark, 2012). Despite these promising factors, there have been no studies to date examining the effectiveness of music as a therapeutic intervention with children and youth with PDOC.

## Music and PDOC populations

Music can be particularly salient as a stimulus depending on the patient's exposure to music. The factors of saliency (Magee and O'Kelly, 2015) and familiarity (van den Bosch et al., 2013) position music as an ideal stimulus for attaining optimum arousal (O'Kelly et al., 2013) and eliciting awareness (Rosenfeld and Dun, 1999) in children and youth with PDOC. Recent guidelines advise that evaluative stimulation used with patients with PDOC should maximize stimuli that are familiar to the patient, highlighting the use of the patient's favorite music (Royal College of Physicians, 2013). One study comparing auditory conditions with varying levels of saliency showed that music with known saliency improved arousal and attention in vegetative state (VS) patients and revealed indications of improved discriminative attention in minimally conscious patients (MCS) (O'Kelly et al., 2013). This study examined physiological and electroencephalography (EEG) responses in patient and healthy cohorts during five auditory conditions: silence, patient-preferred salient music, improvised music entrained to the subject's breathing, patient disliked music, and white noise. The findings suggest that patient responsiveness to different musical conditions is comparable to responsiveness in healthy subjects: both patient and healthy cohorts show a range of significant responses corresponding to arousal and attention during preferred music i.e., music with personal saliency. Healthy responses included globally enhanced EEG power spectra across frequency bandwidths with distinct discriminatory responses across the frontal and temporal regions. During exposure to patient preferred music, frontal alpha frequencies peaked in the MCS patient cohort, and frontal midline theta increased during the preferred musical condition for VS and MCS cohorts. Familiarity is a key factor in inducing emotional responses: Pereira et al. (2011) found that familiar music triggered greater emotion-related brain activity than music that was “liked” but not necessarily “familiar.”

These findings reflect research with other adult neurologically impaired populations such as dementia, Alzheimer's and stroke, where music was shown to improve attention and memory functioning (Särkämö et al., 2013; Baird and Samson, 2014). These two cognitive processes are targeted when assessing and treating people with PDOC. The evidence regarding the use of salient stimuli with people with PDOC supports the notion that memory systems are activated in people with PDOC. However, less is known about the effects of music therapy with children with PDOC. This lack of research is surprising given music's role in healthy development (Trehub, 2005) as well as its widespread use in therapy and education for youth with developmental challenges.

A detailed understanding of the neural effects of music on children and youth with PDOC is lacking due to the cost and technical difficulties in conducting imaging studies with these patients (Ashwal, 2013). Therefore, some assumptions must be made drawing from knowledge about music with non-brain damaged populations. Music is used as a meaningful stimulus with children with normal development to activate emotional responses, to prime cognitive responses, and to facilitate cross-modal learning (Trehub, 2005; Miendlarzewska and Trost, 2013). It recruits neural activity from the early stages of development. Behavioral, physiological, and neural responses to music and vibroacoustic stimuli have been reported in utero, in premature infant and in neonate populations (Shetler, 1990; Woodward et al., 1992; Panthuraamphorn, 1993). Music has also been shown to activate memory function in newborns through recognition tests of familiar music following a two-week delay (Ilari and Polka, 2006). The ubiquitous use of music in human development implies an innate salience to music as a principal activating stimulus and suggests that it has strong ecological validity as a tool for stimulating developing brains. Observations of parent-infant interactions are described in terms of their musical qualities (Stern, 2000). The musical structure of the caregiver's responses supports the development of the infant's brain within this relationship, underpinning later development, and epigenetic expression. There is historical use of musical structure to encode, store, and recall narratives and lessons to pass between generations within social groups (Blacking, 1974). Educators use music and song to aid learning in the classroom in mainstream and specialist schools (Ockelford, 2010), as music can influence learning (Gold et al., 2013). Music is perhaps such a powerful tool for eliciting responses in humans due to its ability to arouse emotions (Blood and Zatorre, 2001) and communicate immediate feeling states from our earliest days of infancy (Malloch and Trevarthen, 2009).

Neurological theories about the nature of consciousness, i.e., theories from anesthesia studies, suggest that consciousness is the complex combination of neural activity in multiple brain areas with the interaction between these brain areas (Vanhaudenhuyse et al., 2011; Di Perri et al., 2014). Musical experience recruits multiple parts of the brain (Bower and Shoemark, 2012; Särkämö et al., 2013) suggesting that it is perhaps an ideal stimulus for maximizing recovery from PDOC. In recent studies researchers have gathered evidence to hypothesize that the pleasure from musical experience is derived from the complex interaction between brain areas known to be involved in cognitive and affective processing (Salimpoor et al., 2013; Zatorre and Salimpoor, 2013). Perhaps music is highly suited to working with both adult and pediatric PDOC patients because of this phenomenon, i.e., that it activates areas of the brain involved in cognitive and emotional processing and that it fosters the interaction between them.

## Future directions

Given its utility in the context of typical development, using music with children with PDOC may be appropriate for a number of reasons. The auditory modality has been established as the most sensitive for diagnosing awareness in adults in vegetative state (Gill-Thwaites and Munday, 1999). The prevalence of visual impairment in pediatric PDOC populations (Huo et al., 1999) suggests that auditory stimulation may be more appropriate in assessment and intervention. Musical stimulation, therefore, has the potential to meet a child's developmental needs and match his/her abilities regardless of language development, sensory impairment, or communication disorder stemming from profound damage. Music provides a powerful tool that is well suited to the needs of children with PDOC for sensory stimulation. It can be individually tailored and offers a powerful stimulus for maximizing the patient's potential to respond (Bower et al., 2014). Music therapy has been shown to provide some degree of benefit in improving consciousness (Meyer et al., 2010). However, the paucity of clinical research warrants further empirical inquiry in order to develop an evidence base for using music as a stimulus in assessment and intervention with pediatric PDOC.

## Author contributions

All authors listed, have made substantial, direct and intellectual contribution to the work, and approved it for publication.

### Conflict of interest statement

The authors declare that the research was conducted in the absence of any commercial or financial relationships that could be construed as a potential conflict of interest.
